# Efficacy and Prognostic Factors for Y-90 Radioembolization (Y-90) in Metastatic Neuroendocrine Tumors with Liver Metastases

**DOI:** 10.1155/2020/5104082

**Published:** 2020-11-23

**Authors:** Erica S. Tsang, Jonathan M. Loree, Janine M. Davies, Sharlene Gill, David Liu, Stephen Ho, Daniel J. Renouf, Howard J. Lim, Hagen F. Kennecke

**Affiliations:** ^1^Division of Medical Oncology, BC Cancer, Vancouver, BC, Canada; ^2^Department of Radiology, Vancouver General Hospital, Vancouver, BC, Canada; ^3^Floyd and Delores Jones Cancer Institute, Virginia Mason Cancer Institute, Seattle, WA, USA

## Abstract

**Background:**

Yttrium-90 (Y-90) can be an effective liver-directed therapy for patients with metastatic neuroendocrine tumors (NETs), but population-based data are limited. We characterized the use of Y-90 in NET patients and identified factors associated with response.

**Methods:**

We identified 49 patients with metastatic liver-dominant NETs across BC Cancer's six regional centres who received Y-90 between June 2011 and January 2017 in British Columbia, Canada. Baseline characteristics, radiographic responses, and outcomes were summarized.

**Results:**

Of the 49 patients who received Y-90, the median age was 56 years (range 21–78), 49% were male, and 94% had an ECOG performance status of 0–1. The primary location of the NET included pancreas (31%), small bowel (41%), large bowel (6%), unknown (14%), and others (12%). 69% of these patients had liver metastases alone, and tumors were graded as *G*1 (61%), *G*2 (25%), *G*3 (2%), and unknown (12%). Prior therapies included surgery (63%), local ablative therapy (25%), somatostatin analogue (69%), and systemic therapy (35%). The median Y-90 dose was 2.2 GBq (range 0.8–3.6), as SIR-spheres (69%) or TheraSpheres (29%). Median time to Y-90 from diagnosis of metastases measured 1.54 years. 88% received segmental Y-90, with 1 (69%), 2 (29%), and 3 (2%) treatments. Y-90 resulted in partial response (53%), stable disease (33%), and progressive disease (12%). Y-90 was well-tolerated, with infrequent grade 3-4 biochemical toxicities (2%) and grade 3 abdominal pain (6%). Longer overall survival (OS) was associated with resection of primary tumor, well-differentiated histology, and low Ki-67. Median OS was 27.2 months (95% CI 8.0–46.5).

**Conclusions:**

In our population-based cohort, Y-90 was well-tolerated in patients with metastatic liver-dominant NETs. Prior surgical resection was an important predictor of OS.

## 1. Introduction

Neuroendocrine tumors (NETs) are rare tumors that have increased in incidence over the last few decades, from 1.09 to 6.98 per 100,000 individuals from 1973 to 2012 [1]. A majority of patients (40–90%) with NETs are diagnosed with liver metastases at presentation, yielding inferior survival outcomes [2]. Median overall survival (OS) for metastatic NETs ranges from 5–57 months [3]. Management options for liver metastases from metastatic NETs include surgery, systemic therapy, or local therapy [4]. Yttrium-90 (Y-90) radioembolization can be an effective local therapy and is recommended in guidelines published by the North American Neuroendocrine Tumor Society and the European Neuroendocrine Tumor Society [5, 6]. While blood supply for normal liver tissue is largely derived from the portal vein, liver metastases from NETs rely on the hepatic artery and are highly vascular. Y-90 radioembolization is a targeted technique to deliver treatment to liver metastases while sparing normal local tissue [7].

However, data are limited regarding the real-world use and efficacy of Y-90 for liver metastases in patients with metastatic NETs. Reported studies to date have been limited by sample size, and outcomes have been variable [8].

In this population-based study, the objectives were to characterize the use of Y-90 radioembolization in patients with metastatic liver-dominant NETs and to determine predictors of efficacy and factors associated with improved survival outcomes.

## 2. Materials and Methods

### 2.1. Study Design and Population

BC Cancer is the provincial institution responsible for cancer therapies for the approximately 4.6 million residents of British Columbia, Canada. Distributed across six centres around the province, BC Cancer manages cancer therapy guidelines, radiation therapy, and funding for all cancer treatments for patients residing in the province, including systemic and local therapies, allowing population-based assessments of outcomes. The BC Cancer Provincial Pharmacy distributes these funded systemic and local therapies across the province.

Y-90 was initially funded for provincial use for patients with advanced, liver-dominant, progressive NETs on June 1, 2011. There is a defined protocol entitled GIYT (http://www.bccancer.bc.ca/health-professionals/clinical-resources/chemotherapy-protocols/gastrointestinal) with prespecified eligibility criteria for Y-90 therapy. Patients with hepatic metastatic NETs and definable disease burden by imaging criteria and mesenteric vascular anatomy amenable to Y-90 are eligible. Exclusion criteria include presence of ascites or encephalopathy, infiltrative disease greater than 50%, extrahepatic disease that is life-limiting and a life expectancy of less than 3 months, and compromised hepatic function (total bilirubin ≥ 2.5 x upper limit of normal (ULN), albumin <3 g/dL, and ALT, AST, or ALP > 5 x ULN. Furthermore, all cases were reviewed at a multidisciplinary liver tumor board with involvement from radiology, interventional radiology, hepatology, medical oncology, and radiation oncology. This was required for BC Cancer approval to proceed with provincially funded Y-90 therapy.

All patients with metastatic liver-dominant NETs who received Y-90 between June 1, 2011, and January 1, 2017, were identified from the BC Cancer Provincial Pharmacy database. TNM classification was performed using the 7^th^ edition of the American Joint Classification on Cancer (AJCC) TNM staging system [9]. Patient demographics, disease characteristics, treatment details, baseline biochemistry, and survival outcomes were extracted by retrospective chart review. Treatment guidelines in place during the time of the study recommended somatostatin analogue therapy only for patients with symptomatic, functional tumors in the advanced setting and not for asymptomatic patients. Treatment response was evaluated based on the Response Evaluation Criteria in Solid Tumors (RECIST) 1.1 criteria [10]. CT imaging was obtained approximately 3 months after the procedure to measure response. Toxicities were assessed using the National Cancer Institute Common Terminology Criteria for Adverse Events (NCI CTCAE) version 4.0 [11]. This study was approved by the BC Cancer Research Ethics Board.

### 2.2. Statistical Analyses

Baseline demographics, clinicopathologic characteristics, and treatment-related toxicities were summarized using descriptive statistics. OS was calculated from both the date of diagnosis and the date of initiation of Y-90 radioembolization. Univariable and multivariable Cox regression and logistic analyses were conducted to explore factors associated with improved response rates and survival outcomes. OS was estimated using the Kaplan–Meier method. All tests were two-sided, with *p* < 0.05 as the cutoff for statistical significance. SPSS version 22.0 was used for statistical analyses (SPSS, Armonk, NY).

## 3. Results

Between June 2011 and January 2017, 49 sequential patients received Y-90 radioembolization at a BC Cancer centre. Median age of diagnosis was 56 years (range 20.7–78.1), and 24 patients (49%) were male. ECOG performance status at time of receipt of Y-90 treatment was 0–1 in 46 of the patients (94%). The location of the primary NET included pancreas (31%), small bowel (41%), large bowel (6%), unknown (14%), and others (8%). The majority of our cohort demonstrated lower grade disease, with grade 1 (61%), grade 2 (25%), grade 3 (2%), and unknown (12%). 69% of patients had only liver metastases. Prior therapies included surgical resection of the primary tumor (63%), local ablative therapy (25%), somatostatin analogue (69%), and systemic therapy (35%).

The median Y-90 dose was 2.2 GBq (range 0.8–3.6), delivered as SIR-spheres (69%) and TheraSpheres (29%). The median time to initiation of Y-90 treatment from diagnosis of metastases measured 1.54 years. Most patients received segmental Y-90 (88%), with 1 (69%), 2 (29%), and 3 (2%) treatments. Patients who received bilobar treatment were all treated early on in this cohort, as bilobar therapy has not been given since 2015 in favor of treating only the regions of tumor progression and minimizing treatment toxicity. Y-90 therapy demonstrated partial response (53%), stable disease (33%), and progressive disease (12%). There were no complete responses in our cohort. Baseline characteristics are detailed in Table 1.

**Figure 1:**
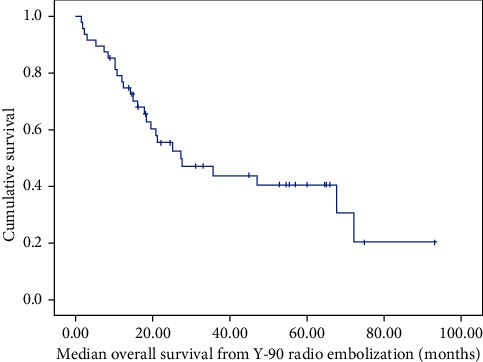
Kaplan–Meier curve of overall survival from initiation of Y-90 radioembolization.

**Figure 2:**
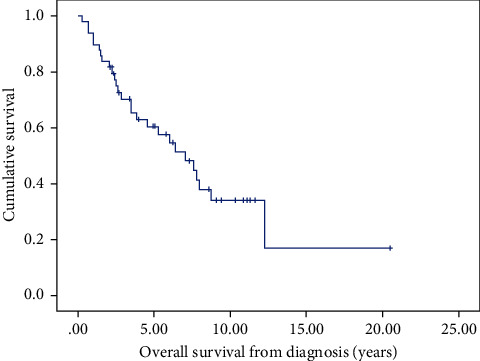
Kaplan–Meier curve of overall survival from date of diagnosis.

Median OS from Y-90 radioembolization measured 27.2 months (95% CI 8.0–46.5; Figure 1), while median OS from diagnosis measured 7.06 years (95% CI 4.3–9.8; Figure 2). Y-90 was generally well-tolerated, with infrequent grade 3-4 biochemical toxicities (2%) and grade 3 abdominal pain (6%). Grade 1 toxicities included fatigue (4%), gastric ulceration (2%), and odynophagia (2%). Biochemical parameters and clinical toxicities are included in Table 2.

On univariable analysis, longer OS from time of Y-90 treatment was associated with previous resection of primary tumor, well-differentiated histology, and low Ki-67 (Table 3). The small sample size and low number of events precluded a meaningful multivariable analysis to confirm this observation of association. In terms of response rate, there were no associated baseline clinicopathologic characteristics, including hepatic tumor burden and tumor differentiation, or previous therapy (all *p* values > 0.05 on logistic regression) (see Table 3).

## 4. Discussion

In this population-based cohort of patients with metastatic liver-dominant NETs, Y-90 treatment was generally well-tolerated with infrequent toxicities. The majority of patients (86%) achieved partial response or stable disease, with only 12% demonstrating disease progression. Prior surgical resection was associated with longer OS from the time of Y-90 treatment, while there were no baseline clinicopathologic characteristics that seemed to be predictive of response rate. To our knowledge, this study represents the first Canadian perspective on real-world efficacy and tolerability of Y-90 in patients with metastatic liver-dominant NETs. At present, there are limited data in the literature regarding the use and efficacy of Y-90, and this study contributes to the collective experience.

In our study, we found that 53% patients achieved a partial response and median OS from Y-90 radioembolization measured 27.2 months. These response rates and outcomes are similar to previously reported response rates, which ranged from 22–63%, and median survival, which measured from 22–70 months [4]. Toxicities were also comparable between studies, with a 2% incidence of grade 3-4 biochemical toxicities and 6% incidence of grade 3 abdominal pain. Rhee et al. reported a 14% rate of grade 3-4 toxicities, while other groups such as Kennedy et al. found 6.5-33% grade 3 toxicity [12, 13]. As summarized in the guidelines from the NET-Liver-Metastases Conference, much of these data are derived from small retrospective studies rather than level I data [7]. This emphasizes the need for expanding our shared experience with Y-90 in advanced liver-dominant NETs and for greater collaboration.

In our univariable analysis, prior surgical resection, well-differentiated histology, and low Ki-67 were associated with longer OS from the time of Y-90 treatment (see Table 3). No baseline characteristics were found to be significantly associated with response rate to Y-90 on logistic regression. Extrahepatic metastases, prior progression on systemic therapy, and multiple courses of Y-90 have all been reported to be prognostic factors for survival in this patient population. A majority of these previous studies are also limited by small sample sizes, so this may be why the effect was not seen in our cohort. Singla et al. presented a study examining the correlation between Ki-67 and benefit with Y-90, reporting that patients with Ki-67 score ≥3% may derive a greater benefit with Y-90 [14]. Ki-67 measured ≥3% in 36% of our patients, but was not a prognostic marker in our analysis. This may be related to the small sample sizes in both studies. There were 44 patients treated with Y-90 in their study with Ki-67 available in 64% of cases, while our cohort totaled 49 patients. There are also novel techniques that may play a role in prognostication, such as the ^68^Ga-DOTATOC scan, which now has an established role in the management of neuroendocrine malignancies. In a small study, Filippi et al. employed the ^68^Ga-DOTATOC scan to determine molecular response and demonstrated that molecular response seen on the ^68^Ga-DOTATOC scan was associated with improved OS [15].

The retrospective nature and limited sample size of our study precludes multivariable analyses for predictive markers of response rate. In addition, the sample size limits the generalizability of our data, but it is perhaps reassuring that the response rates, survival outcomes, and tolerability remain comparable between the small retrospectives studies that are currently available in the literature. Prospective multicentre trials exploring associations between patient and pathologic factors with response rates and outcomes may help us in risk stratification and to better select eligible patients who are most likely to benefit from Y-90 radioembolization. This descriptive study helped to share our provincial data and outcomes.

## 5. Conclusion

In this population-based cohort, Y-90 was well-tolerated in patients with metastatic liver-dominant NETs. Previous surgical resection was an important predictor of OS from time of Y-90 treatment. Prospective collaborative cohorts will be helpful in better delineating patient selection criteria to ensure that patients derive the maximal benefit from Y-90 radioembolization.

## Figures and Tables

**Table 1:** Baseline demographic and clinicopathologic characteristics of patients with metastatic liver-dominant NETs who received Y-90 radioembolization

Baseline characteristic	Number of patients (%)
*Age at diagnosis of metastatic disease (years)*	
Median (range)	56.0 (20.71–78.14)

*Gender*	
Male	**24 (49%)**
Female	25 (51%)

*ECOG status at Y-90 treatment*	
0–1	46 (94%)
≥2	2 (4%)
Unknown	1

*Location of primary NET*	
Pancreas	15 (31%)
Small bowel	20 (41%)
Large bowel	3 (6%)
Unknown	7 (14%)
Other	4 (8%)

*Grade*	
1	30 (61%)
2	12 (25%)
3	1 (2%)
Unknown	6 (12%)

*Ki-67 (%)*	
≤2	18 (37%)
3–20	17 (34%)
>20	1 (2%)
Unknown	13 (27%)

*Mitotic count (/hpf)*	
<2	23 (47%)
2–20	13 (27%)
>20	0
Unknown	13 (27%)

*Baseline chromogranin A*	
Median (range)	325 (7–44100)

*Hepatic tumor burden*	
<33%	9 (18%)
33–66%	32 (65%)
>66%	7 (14%)
Unknown	1 (2%)

*Number of extrahepatic metastases*	
0	34 (69%)
1	11 (22%)
2	3 (6%)
3	1 (2%)

*Previous therapies*	
Surgical resection of primary tumor	31 (63%)
Local ablative therapy	12 (25%)
SSA	34 (69%)
Systemic therapy	17 (35%)

*Dose of Y-90 (GBq)*	
Median (range)	2.2 (0.80–3.60)

*Type of Y-90*	
TheraSpheres	14 (29%)
SIR-Spheres	34 (69%)
Unknown	1 (2%)

*Type of treatment*	
Liver segment	43 (88%)
Whole liver	5 (10%)
Unknown	1 (2%)

*Response to Y-90 radioembolization*	
Complete response	0
Partial response	26 (53%)
Stable disease	16 (33%)
Progressive disease	6 (12%)
Unknown	1 (2%)

*Number of liver segments treated*	
1	9 (18%)
2	31 (63%)
3	4 (8%)
4	3 (6%)
Unknown	2 (4%)

*Number of Y-90 treatments*	
1	34 (69%)
2	14 (29%)
3	1 (2%)

**Table 2:** Biochemical and clinical toxicities after Y-90 radioembolization, evaluated at 3 months posttreatment.

Biochemical and clinical toxicities	Number of patients (%)
*ALT (U/L)*	
Grade 1	2 (4%)
Grade 2	0
Grade 3	1 (2%)
Grade 4	0

*AST (U/L)*	
Grade 1	11 (22%)
Grade 2	1 (2%)
Grade 3	1 (2%)
Grade 4	0

*ALP (U/L)*	
Grade 1	26 (53%)
Grade 2	3 (6%)
Grade 3	0
Grade 4	1 (2%)

*WBC*	
Grade 1 toxicity	4 (8%)
Grade 2 toxicity	1 (2%)
Grade 3	0
Grade 4	0

*Bilirubin (μmol/L)*	
Grade 1 toxicity	5 (10%)
Grade 2 toxicity	0
Grade 3	0
Grade 4	0

*Clinical toxicities*	
Fatigue, grade 1	2 (4%)
Abdominal pain, grade 3	3 (6%)
Gastric ulceration, grade 1	1 (2%)
Odynophagia, grade 1	1 (2%)

AST = aspartate aminotransferase; ALT =  alanine aminotransferase; ALP = alkaline phosphatase.

**Table 3:** Univariable analysis of factors associated with improved median overall survival.

	Univariable	
	HR (95% CI)	*p* value
Age (continuous)	1.01 (0.98–1.04)	0.59

Gender		0.88
Female	Reference (1.00)	
Male	1.06 (0.50–2.28)	

ECOG at Y-90 treatment		
0	Reference (1.00)	
1	1.64 (0.66–4.13)	0.29
2	1.25 (0.15–10.42)	0.84

Tumor differentiation		
Well	Reference (1.00)	
Moderate	2.23 (0.89–5.59)	0.09
Poor	**13.29 (1.42–124.41)**	**0.02**

Ki67		
≤2	Reference (1.00)	
3–20	1.95 (0.77–4.79)	0.16
>20	15.56 (1.50–160.98)	**0.02**
Extrahepatic metastases	2.14 (0.97–4.71)	0.06

Hepatic tumor burden		
<33%	Reference (1.00)	0.46
33–66%	2.01 (0.60–6.78)	0.26
>66%	1.32 (0.26–6.60)	0.74
Baseline CgA (continuous)	1.00	0.16
Previous local therapies	0.79 (0.33–1.88)	0.59
Previous SSA	0.87 (0.39–1.96)	0.74
Previous systemic therapies	1.56 (0.72–3.37)	0.26
Previous surgery	**0.42 (0.19–0.91)**	**0.03**

HR = hazard ratio; ECOG = Eastern Cooperative Oncology Group performance status; CgA = chromogranin A; SSA = somatostatin analogue.

## Data Availability

The data used to support the findings of this study are available from the corresponding author upon request.
